# Utilization of food outlets and intake of minimally processed and ultra-processed foods among 7 to 14-year-old schoolchildren. A cross-sectional study

**DOI:** 10.1590/1516-3180.2017.0211061217

**Published:** 2018-03-29

**Authors:** Elizabeth Nappi Corrêa, Anabelle Retondario, Mariane de Almeida Alves, Liliana Paula Bricarello, Gabriele Rockenbach, Patrícia de Fragas Hinnig, Janaina das Neves, Francisco de Assis Guedes de Vasconcelos

**Affiliations:** I PhD. Dietitian and Assistant Professor, Department of Nutrition, Universidade Federal de Santa Catarina (UFSC), Florianópolis (SC), Brazil.; II MSc. Dietitian and Doctoral Student, Universidade Federal de Santa Catarina (UFSC), Florianópolis (SC), Brazil.; III MSc. Dietitian, Universidade Federal de Santa Catarina (UFSC). Florianópolis (SC), Brazil.; IV MSc. Dietitian and Doctoral Student, Universidade Federal de Santa Catarina (UFSC), Florianópolis (SC), Brazil.; V PhD. Dietitian and Assistant Professor, Department of Nutrition, Universidade Federal de Santa Catarina (UFSC), Florianópolis (SC), Brazil.; VI PhD. Dietitian and Assistant Professor, Department of Nutrition, Universidade Federal de Santa Catarina (UFSC), Florianópolis (SC), Brazil.; VII PhD. Dietitian and Associate Professor, Department of Nutrition, Universidade Federal de Santa Catarina (UFSC), Florianópolis (SC), Brazil.; VIII PhD. Dietitian and Professor, Universidade Federal de Santa Catarina (UFSC), Florianópolis (SC), Brazil.

**Keywords:** Child, Adolescent, Environment and public health

## Abstract

**BACKGROUND:**

Access to food retailers is an environmental determinant that influences what people consume*.* This study aimed to test the association between the use of food outlets and schoolchildren’s intake of minimally processed and ultra-processed foods.

**DESIGN AND SETTING:**

This was a cross-sectional study conducted in public and private schools in Florianópolis, state of Santa Catarina, southern Brazil, from September 2012 to June 2013.

**METHODS:**

The sample consisted of randomly selected clusters of schoolchildren aged 7 to 14 years, who were attending 30 schools. Parents or guardians provided socioeconomic and demographic data and answered questions about use of food outlets. Dietary intake was surveyed using a dietary recall questionnaire based on the previous day’s intake. The foods or food groups were classified according to the level of processing. Negative binomial regression was used for data analysis.

**RESULTS:**

We included 2,195 schoolchildren in the study. We found that buying foods from snack bars or fast-food outlets was associated with the intake frequency of ultra-processed foods among 11-14 years old in an adjusted model (incidence rate ratio, IRR: 1.11; 95% confidence interval, CI: 1.01;1.23). Use of butchers was associated with the intake frequency of unprocessed/minimally processed foods among children 11-14 years old in the crude model (IRR: 1.11; 95% CI: 1.01;1.22) and in the adjusted model (IRR: 1.11; 95% CI: 1.06;1.17).

**CONCLUSIONS:**

Use of butchers was associated with higher intake of unprocessed/minimally processed foods while use of snack bars or fast-food outlets may have a negative impact on schoolchildren’s dietary habits.

## INTRODUCTION

Ultra-processed foods are characterized by high energy density and higher content of free sugars, sodium and total and saturated fat, combined with lower protein and fiber content.^1^ They have hyper-palatability, are often offered in large-sized portions and are accompanied by aggressive marketing strategies, which leads to overeating.^2^ There is evidence that increased intake of ultra-processed foods and reduced intake of unprocessed or minimally processed foods, and concomitant increases in rates of inactivity, are the factors most strongly associated with obesity and other non-communicable chronic diseases.^3^

In high-income countries, consumption of ultra-processed foods has been widespread for a long time. In middle and low-income countries, there is a rapidly-growing tendency to replace unprocessed and minimally processed foods and meals prepared by traditional culinary methods with commercial ready-to-eat products. This has occurred mainly since the 1980s.^4^ The typical dietary intake of Brazilian adolescents is based on excessive consumption of ultra-processed foods.^5^

Changes in eating habits are widely attributed to environmental characteristics that encourage excessive nutritional intake.^6^ The current dietary intake and physical activity profile is partially a result of environmental factors, and particularly the features of the built environment,^7^ i.e. the environment created by modern societal trends.^8^

Industrialization, urbanization, economic development and globalized markets have created an environment with great availability and accessibility of high levels of energy-dense foods. Furthermore, there is intense social pressure on people to eat these foods.^9^

Residents of communities with easy access to foods that are considered to be healthy tend to have healthier diets.^10^ The availability and accessibility of foods that are considered to be either healthier or less healthy are important factors that influence the eating habits of adolescents and adults.^9^

One of the most important environmental determinants influencing people’s diets is their degree of access to the establishments that sell food in their neighborhoods.^10^ But a significant proportion of studies investigating food environments only evaluate the availability of establishments that sell food and do not consider whether people actually use them.^11^

Given that features of the environment can influence people’s nutritional choices, this study aimed to investigate the association between use of food outlets and intake of minimally processed and ultra-processed foods among 7 to 14-year-old schoolchildren.

## METHODS

This was a cross-sectional analytical study conducted in the city of Florianópolis, capital of the state of Santa Catarina, in southern Brazil.

The procedures that were used for sample size calculation and sampling have been described elsewhere.^12^ Briefly, we had a possible sample of 2,506 schoolchildren, stratified according to municipal administrative districts, type of school (public or private) and age group (7-10 or 11-14 years). We randomly selected 30 schools (19 public and 11 private schools), and schoolchildren from each school through clusters. Trained researchers collected data between September 2012 and June 2013.

Seven to 14-year-old schoolchildren were recruited after their guardians had signed free and informed consent forms. Individuals with disabilities that prevented assessment and pregnant adolescents were excluded. Socioeconomic, demographic and anthropometric data and information on the dietary intakes and lifestyles of these schoolchildren and their families were acquired through direct measurement or administration of specific data collection instruments, as described below.

The project was approved by the Human Research Ethics Committee at the Federal University of Santa Catarina (no. 120341/2012).

### Socioeconomic and demographic data and use of food outlets 

The children’s parents received a questionnaire covering socioeconomic and demographic information about the schoolchildren and their families (sex, age and mother’s educational level) and data on where they lived and what type of food outlets they used.

Data on food outlets and their use by schoolchildren and their families were obtained through a questionnaire in which the outlets were categorized into eight types: restaurants, snack bars/fast-food outlets, street vendors, supermarkets, mini-markets, greengrocers/public markets, bakeries and butchers. The children’s parents answered “yes” if they frequently used any of the food outlets or “no” if they did not.

Socioeconomic status was estimated based on the average income for the weighting area where the schoolchildren lived (each weighting area comprised a cluster of census tracts).^13^ This information was obtained from the 2010 Brazilian demographic census, published by the Brazilian Institute for Geography and Statistics (IBGE).^13^ The income values were given in reais (R$) by IBGE, but for the purposes of this study they were converted into dollars (USD) using the mid-price for the dollar in August 2010 (1 USD = R$ 1.75), when data collection for the census began.

### Dietary intake assessment and NOVA food classification

Data on dietary intake were obtained from a single dietary recall questionnaire based on the previous day’s intake, known as QUADA-3 (Questionário Alimentar do Dia Anterior). This tool was administered at each school, directly to the children, because the tool was developed specifically for children to be able to understand it. It consists of an illustrated questionnaire covering six eating occasions (breakfast, mid-morning snack, lunch, mid-afternoon snack, dinner, and late-evening snack) in chronological order and includes 21 foods or food groups for each eating occasion. This tool was structured as a record in which the frequencies of consumption of different types of food over the previous day were ascertained, but not the quantities.^14^

This questionnaire was given to the schoolchildren and, with help from the researchers, they indicated which foods they had eaten on the previous day at each of these mealtimes. Because the tool was applied within the school setting and there was no school on Saturdays and Sundays, it was not possible to obtain data representing food consumption for Fridays and Saturdays. By giving the questionnaire to the participating schools on alternate school days, we were able to get information on dietary intake on a selection of different days of the week (including Sundays). For the purposes of this study, foods covered by QUADA-3^14^ were classified in accordance with the level of processing, as proposed by the NOVA food classification.^4^

The NOVA classification is based on types of food processing, and food groups, according to the extent and purpose of the processing to which they have been subjected.^4^ NOVA describes four categories for food classification, according to processing:

1. unprocessed and minimally processed foods;
2. processed culinary ingredients;
3. processed foods; and
4. ultra-processed foods.^4^

Based on the NOVA definitions, the categories were established as described in the following. Unprocessed foods were considered to be edible parts of animals, plants, fungi or algae that are in the same state as when they were extracted from nature. Minimally processed foods were considered to be unprocessed foods that had been modified through minimal processing after extraction from nature, such as pasteurization, drying, boiling and other processes that do not involve addition of other substances. Processed foods were considered to be those made by adding cooking ingredients to unprocessed and minimally processed foods, for example, fruit and vegetable preserves, salted seeds and smoked meats. Finally, ultra-processed foods were considered to be manufactured preparations containing long lists of ingredients, many of which are utilized exclusively by the industry, such as casein, lactose and hydrogenated fats.^4^ Processed cooking ingredients were not included in the categories that were set up, because we were dealing with meals, and not with ingredients (such as salt, sugar, honey, oils and fats).

For the analysis, eight of the 21 foods and food groups investigated using QUADA-3^14^ were defined as ultra-processed foods: yoghurt, chocolate milk, “treats” (candies, lollipops, biscuits with fillings and ice cream), potato crisps (US: chips) or chips (US: French fries), sugary soft drinks, pizzas, hamburgers, breads and biscuits. The decision to classify bread as ultra-processed food was justified because our tool was not able to specify what type of bread was consumed and, according to the NOVA classification, breads are ultra-processed if they have other ingredients besides wheat, yeast, water and salt.^4^ In this way, we follow the adaptation proposed by Bielemann et al.^15^ Twelve foods and food groups were defined as unprocessed or minimally processed foods: vegetables, leafy greens, fruit, fruit juice, rice, beans, pasta, vegetable soup, red meat, chicken, fish and seafood, milk and coffee with milk. The processed foods category was excluded because only one food (cheese) belonged to this group.

### Statistical treatment

The data were processed using EpiData 3.0, with data quality controlled by means of double entry. Statistical analyses were conducted using STATA version 13.0 (StataCorp, Texas, USA), considering the design effect in all analyses.

Data were expressed using absolute and relative frequencies. Associations between exposures (use of each food outlet) and outcomes (the frequencies at which ultra-processed and unprocessed/minimally processed foods were consumed during the previous day) were assessed using negative binomial regression analysis for count data with the presence of excess zeros. Initially, a crude analysis model was constructed. The multivariate model was adjusted for sex, mother’s educational level (≤ 8 years or > 8 years) and the income of the weighting area (expressed in terciles). Stratified analysis was conducted according to age (7-10 or 11-14). A significance level of P < 0.05 was used.

## RESULTS

The characteristics of the study population are shown in Table 1. Of the 2,506 schoolchildren, 311 were excluded because lack of an address or its incompleteness prevented their identification. This left 2,195 children (88% of the total sample) available for inclusion. Among the schoolchildren, there were slight majorities of girls (52%), students aged 7-10 years (60%), students enrolled at public schools (61%) and students whose mothers had had at least 8 years of schooling (75%).

**Table 1:** Distribution of sociodemographic characteristics among 7 to 14-year-old schoolchildren. Florianópolis, Brazil, 2012-2013

Sociodemographic characteristics	n (%)
Sex	
Female	1149 (52.3)
Male	1046 (47.7)
Age group (years)	
7-10	1307 (59.5)
11-14	888 (40.5)
Type of school	
Public	1346 (61.3)
Private	849 (38.7)
Mother’s educational level^a^	
≤ Completion of primary education (≤ 8 years)	540 (25.3)
≥ Started secondary education (> 8 years)	1595 (74.7)
Income for weighting area (US dollars - USD)*	
3^rd^ tercile (3,022 - 6,165 USD)	669 (30.5)
2^nd^ tercile (2,181 - 3,021 USD)	811 (36.9)
1^st^ tercile (1,395 - 2,180 USD)	715 (32.6)

^a^n = 2,135; *Conversion from Brazilian Real using the mid-price for the dollar in August 2010 (1 USD = R$ 1.75).

Table 2 shows the absolute and relative frequencies of the schoolchildren’s dietary intake of each type of food, classified as either “unprocessed/minimally processed” or “ultra-processed”. Approximately 99% of the schoolchildren reported that they had eaten both unprocessed/minimally processed foods and ultra-processed foods on the day before the survey. In the unprocessed/minimally processed group of foods, more than half of the children had eaten rice, red meat or chicken, and beans. Among the ultra-processed foods, more than half of the sample had eaten bread or biscuits, sugary soft drinks and chocolate milk.

**Table 2:** Dietary intake on previous day, classified according to foods and food groups and their degree of processing, among 7 to 10-year-old and 11 to 14-year-old schoolchildren. Florianópolis, Brazil, 2012-2013

Foods and food groups^**a**^	Eaten the previous day n (%)	
	7 to 10-year-old schoolchildren	11 to 14-year-old schoolchildren
Unprocessed/minimally processed foods	1,333 (99.5)	840 (98.7)
Rice	999 (74.6)	651 (76.7)
Red meat or chicken	821 (63.0)	604 (70.4)
Beans	848 (63.0)	482 (56.7)
Fruit	688 (51.3)	389 (45.0)
Pasta	664 (48.6)	380 (44.9)
Fruit juice	582 (43.8)	343 (41.5)
Coffee with milk	496 (35.8)	330 (37.2)
Milk	353 (26.1)	209 (23.9)
Vegetables	272 (21.1)	196 (24.3)
Leafy greens	254 (19.9)	204 (24.4)
Vegetable soup	234 (16.2)	57 (6.4)
Fish or seafood	178 (12.4)	97 (10.7)
Ultra-processed foods	1,323 (98.4)	834 (97.6)
Bread or biscuits	1,090 (80.4)	700 (80.9)
Sugary soft drinks	797 (58.8)	497 (59.0)
Chocolate milk	759 (55.9)	422 (49.6)
Treats	603 (45.8)	402 (46.2)
Yoghurt	449 (32.9)	234 (27.2)
Pizzas or hamburgers	328 (23.5)	177 (21.0)
Chips (US: French fries)	255 (17.8)	157 (19.1)
Crisps (US: chips)	235 (16.7)	149 (17.5)

^a^The category identified as “processed food” was excluded because only one food (cheese) belonged to this group.

Table 3 lists the prevalence of use of food outlets and the association between use of food outlets and intake of ultra-processed foods on the previous day. The food outlets that were most frequented were supermarkets (96.12%), public markets (88.80%) and bakeries (87.76%). We noted that use of snack bars and fast-food outlets had a positive association with intake of ultra-processed foods in the adjusted model among the children aged 11-14 years (incidence rate ratio, IRR: 1.11; 95% confidence interval, CI: 1.01;1.23).

**Table 3:** Association between frequency of ultra-processed food intake and types of food outlet frequented by the family among 7 to 10-year-old and 11 to 14-year-old schoolchildren. Florianópolis, Brazil, 2012-2013

Variables	Use n (%)	Crude model IRR (95% CI)	P-value^**a**^	Crude model IRR (95% CI)	P-value^**a**^	Adjusted model IRR (95% CI)	P-value^**a**^	Adjusted model IRR (95% CI)	P-value^**a**^
		7 to 10-year-old schoolchildren		11 to 14-year-old schoolchildren		7 to 10-year-old schoolchildren		11 to 14-year-old schoolchildren	
Supermarkets	2,057 (96.12)		0.993		0.939		0.548		0.964
No		1		1		1		1	
Yes		1.00 (0.70;1.45)		0.99 (0.62;1.57)		1.09 (0.73;1.63)		1.01 (0.61;1.65)	
Mini-markets	1,307 (67.83)		0.635		0.486		0.466		0.886
No		1		1		1		1	
Yes		0.98 (0.87;1.10)		1.03 (0.93;1.14)		0.97 (0.87;1.08)		1.01 (0.90;1.12)	
Bakeries	1,785 (87.76)		0.470		0.806		0.566		0.693
No		1		1		1		1	
Yes		1.03 (0.91;1.18)		0.98 (0.73;1.31)		1.03 (0.89;1.20)		0.96 (0.72;1.28)	
Butchers	865 (47.76)		0.106		0.204		0.075		0.276
No		1		1		1		1	
Yes		1.08 (0.97;1.21)		1.08 (0.93;1.26)		1.09 (0.98;1.20)		1.07 (0.91;1.26)	
Public markets	1,839 (88.80)		0.865		0.540		0.956		0.631
No		1		1		1		1	
Yes		0.99 (0.82;1.19)		1.08 (0.75;1.55)		0.99 (0.83;1.19)		1.06 (0.75;1.49)	
Street vendors	1,391 (66.62)		0.218		0.184		0.304		0.158
No		1		1		1		1	
Yes		1.03 (0.97;1.09)		1.06 (0.95;1.17)		1.02 (0.96;1.09)		1.06 (0.96;1.18)	
Snack bars/ fast-food outlets	1,691 (80.26)		0.358		0.114		0.325		0.044
No		1		1		1		1	
Yes		1.05 (0.91;1.21)		1.09 (0.96;1.23)		1.06 (0.91;1.23)		1.11 (1.01;1.23)	
Restaurants	1,772 (83.82)		0.282		0.323		0.066		0.473
No		1		1		1		1	
Yes		1.05 (0.93;1.18)		0.93 (0.78;1.13)		1.08 (0.99;1.18)		0.96 (0.81;1.13)	

^a^Negative binomial regression. NOTES: Crude model: intake of ultra-processed foods and use of food outlets. Adjusted model: crude model + sex, mother’s schooling and income in weighting area. Boldface indicates statistical significance (P < 0.05).

The association between use of food outlets and intake of unprocessed/minimally processed foods is shown in Table 4. An association was found between use of butchers and the frequency of intake of unprocessed/minimally processed foods among the children aged 11-14 years in the crude model (IRR: 1.11; 95% CI: 1.01;1.22) and in the adjusted model (IRR: 1.11; 95% CI: 1.06;1.17).

**Table 4:** Association between frequency of unprocessed/minimally processed food intake and types of food outlet frequented by the family among 7 to 10-year-old and 11 to 14-year-old schoolchildren. Florianópolis, Brazil, 2012-2013

Variables	Use n (%)	Crude model IRR (95% CI)	P-value^**a**^	Crude model IRR (95% CI)	P-value^**a**^	Adjusted model IRR (95% CI)	P-value^**a**^	Adjusted model IRR (95% CI)	P-value^**a**^
		7 to 10-year-old schoolchildren		11 to 14-year-old schoolchildren		7 to 10-year-old schoolchildren		11 to 14-year-old schoolchildren	
Supermarkets	2,057 (96.12)		0.456		0.212		0.076		0.114
No		1		1		1		1	
Yes		1.04 (0.89;1.21)		1.14 (0.88;1.49)		1.10 (0.98;1.24)		1.22 (0.91;1.64)	
Mini-markets	1,307 (67.83)		0.517		0.444		0.635		0.378
No		1		1		1		1	
Yes		1.03 (0.90;1.18)		1.07 (0.83;1.39)		1.03 (0.87;1.21)		1.08 (0.86;1.36)	
Bakeries	1,785 (87.76)		0.328		0.422		0.376		0.512
No		1		1		1		1	
Yes		0.94 (0.80;1.11)		1.10 (0.79;1.54)		0.95 (0.80;1.12)		1.09 (0.74;1.61)	
Butchers	865 (47.76)		0.225		0.040		0.261		0.006
No		1		1		1		1	
Yes		1.07 (0.93;1.24)		1.11 (1.01;1.22)		1.08 (0.91;1.27)		1.11(1.06;1.17)	
Public markets	1,839 (88.80)		0.865		0.728		0.760		0.846
No		1		1		1		1	
Yes		0.98 (0.86;1.12)		0.98 (0.81;1.18)		0.99 (0.86;1.13)		0.99 (0.83;1.18)	
Street vendors	1,391 (66.62)		0.110		0.153		0.421		0.141
No		1		1		1		1	
Yes		0.97 (0.95;1.01)		0.95 (0.88;1.03)		0.98 (0.93;1.04)		0.94 (0.85;1.04)	
Snack bars/ fast-food outlets	1,691 (80.26)		0.566		0.328		0.202		0.435
No		1		1		1		1	
Yes		1.01 (0.98;1.03)		0.94 (0.80;1.11)		1.02 (0.98;1.05)		0.94 (0.76;1.16)	
Restaurants	1,772 (83.82)		0.318		0.688		0.292		0.517
No		1		1		1		1	
Yes		1.04 (0.93;1.17)		0.98 (0.82;1.16)		1.05 (0.93;1.18)		0.96 (0.81;1.14)	

^a^Negative binomial regression. NOTES: Crude model: intake of unprocessed or minimally processed foods and use of food outlets. Adjusted model: crude model + sex, mother’s schooling and income in weighting area. Boldface indicates statistical significance (P < 0.05).

No association was found between use of food outlets and the frequencies of intake of unprocessed/minimally processed foods and ultra-processed foods among the children aged 7-10 years.

## DISCUSSION

Almost all of the schoolchildren interviewed stated that they had eaten ultra-processed foods on the previous day. Eating habits that include excessive consumption of ultra-processed foods are increasing, and this has been attributed to factors such as convenience, simplicity, accessibility and shorter preparation times.^3^ Rises in the incomes of Brazilian families, particularly among those in low-income categories, have also been identified as a factor that increases poorer families’ access to these products.^16^ In association with these factors, ready-to-eat products are marketed aggressively, especially targeting children.^17^

The dietary intake observed in this study was similar to what has been seen in nationwide studies in Brazil. A study conducted in 2016 among 12 to 17-year-old adolescents found that the most frequently consumed unprocessed/minimally processed foods were rice, beans and beef. Among the ultra-processed foods, bread, biscuits and sugary soft drinks had the greatest prevalence of consumption.^5^ Similar dietary behavior was observed by Levy et al.,^18^ who found that the most frequently consumed healthy food item was beans, while unhealthy eating was marked by high intake of sugary soft drinks and treats. The share of the Brazilian diet accounted for by ultra-processed products has been growing since the 1980s in metropolitan areas and the same pattern was confirmed in the 2000s, independently of socioeconomic status.^19^

The findings of the present study suggest that there is a positive association between use of snack bars and fast-food outlets and intake of ultra-processed foods. Few studies had explored the relationship between the intake of foods classified according to the level of processing and use of different food outlets.^20^^,^^21^ Since this subject is relatively new, it is reasonable to compare the present results with those of studies that investigated the availability and density of food outlets and the intake of foods, but not necessarily using classifications according to the level of processing.

A Canadian study reported that living near fast-food outlets had a marked influence on the eating habits of 11 to 15-year-old adolescents. Those in neighborhoods with moderate or high density of fast-food restaurants had an excessive intake of fast food.^22^ Similar findings were reported among Danish adolescents of the same age, among whom the boys who had greater exposure to fast-food restaurants in the environs of their schools had higher intake of the foods sold at these establishments than did those who were not exposed to these restaurants.^20^

Another Canadian study on 810 adolescents aged 11 to 14 found that there was poorer quality of diet, with higher intake of fast foods, among those who lived close to or went to schools close to convenience stores and outlets selling fast foods.^23^ Similar results were reported by Laska et al.,^24^ who found that the sugar-sweetened beverage intake of 349 American adolescents was associated with living close to fast-food restaurants, convenience or grocery stores or any retail facility. Adolescents who lived close to these retailers were also more likely to buy foods from these outlets when their parents or guardians were not around.^24^

Our study showed that there was a positive association between the use of snack bars and fast-food outlets and the intake of ultra-processed foods, when stratified by age, among the adolescents (from 11 to 14 years). Adolescents have more autonomy in relation to purchasing food. In addition to access and autonomy, food choices also reflect an array of personal and social values. Adolescence is an important developmental age accompanied by notable declines in a range of health-related behaviors.^24^

In the present study, use of butchers was associated with higher intake of unprocessed/minimally processed foods, probably because in this type of establishment, fresh meat that needs culinary preparation is sold. However, use of supermarkets was not associated with intake of unprocessed or minimally processed foods. It needs to be borne in mind that supermarkets sell a wide variety of different food items, ranging from fresh and unprocessed foods to ultra-processed foods.^25^

Living at a greater distance from supermarkets in metropolitan areas reduced the intake of fruits and vegetables among the adult population in a nationwide study in the United States.^26^ No associations were observed in another study^23^ that was conducted in London among adolescents (11 to 13 years old). A study conducted in the city of Santos (state of São Paulo, Brazil) showed that using supermarkets to purchase fresh products was associated with lower levels of purchases of ultra-processed foods from the supermarket.^21^ Another study that was developed among 1,721 children aged 9-10 years, in Norfolk, England, found that higher density of supermarkets was associated not only with higher consumption of fruits and vegetables, but also with more sweets, sugary drinks, breakfast cereals and white bread. Increasing distance from convenience and takeaway food stores was associated with lower intake of crisps, chips, sweets, chocolate and white bread.^27^

The cost of foods and its influence on consumption has also been discussed in the literature. The difference in cost between ultra-processed and minimally processed foods in New Zealand supermarkets was studied by Lutein et al.^28^ No statistically significant difference was found in terms of cost, but the authors suggested that ultra-processed products, which made up 83% of the packaged products offered, might have provided time-poor consumers with greater value for money.^28^ Swiss researchers examining the driving forces behind increasing consumption of convenience foods identified 15 drivers for purchases of these foods. Among these forces, age, concern for naturalness, nutritional knowledge and cooking skills were the strongest drivers.^29^

## LIMITATIONS

One limitation of this study relates to the dietary intake questionnaire, which assesses frequency of intake rather than quantity (qualitative method). This eliminates difficulties relating to children’s assessments of portion sizes and simplifies the memory task through only covering foods eaten the previous day.^30^ It is an approach that enables use of a relatively brief questionnaire that is easy for children to answer with minimal assistance.^14^ However, one day’s dietary intake may not be representative of habitual individual intakes. On the other hand, our results are made more robust by the reasonable sample size and the good coverage of different days of the week, including weekdays and Sundays.^14^ Additionally, we recognize that not every type of food purchased from each type of outlet was evaluated.

The strengths of our study were the sample size and the adjustment of the data for sociodemographic and economic control variables. Classification of foods according to degree of processing introduces additional evidence to help explain the global epidemic of non-communicable chronic diseases, including obesity among children and adolescents,^31^ considering that classification of foods according to nutritional categories alone has not explained this relationship.^3^

Many different studies have investigated the availability and presence of food outlets and dietary intake among children and adolescents.^32^^,^^33^ In the present study, parents and their children were asked whether they actually used these establishments, without attempting to identify whether they were located in the vicinities of their schools/homes. Research conducted by Leite et al.^34^ observed that food retailers that mostly sold ultra-processed foods were significantly closer to schools than were those that mostly sold foods with lesser degrees of processing, thus demonstrating the importance of also investigating retailers close to schools.

## CONCLUSION

The results suggest that use of butchers contributed towards increasing the intake of unprocessed/minimally processed foods, thus showing the importance of the choices of places to buy foods. In contrast, schoolchildren’s use of snack bars and fast-food outlets was related to their intake of ultra-processed foods, and this negatively influenced their dietary habits. Future studies should incorporate other features of the environment that influence dietary intake and choices. In addition, mapping of retail food outlets in specific environments could facilitate development of ways to promote healthy eating that are suitable for specific populations and the environments in which they live. Public health actions should address food labeling to make it easier for consumers to understand what they are buying. A means to help direct people towards places that sell fresh and healthy foods so that they avoid outlets for ultra-processed foods is also needed.
